# Development of a Novel Lysosomal Gene-based Prognostic Panel and Uncovering EIF4EBP1 as a Biomarker for Breast Cancer

**DOI:** 10.2174/0113892029357021250626210819

**Published:** 2025-07-03

**Authors:** Bingkun Wang, Nianjin Wei, Meiyu He, Guocai Zhong, Shujun Zhang

**Affiliations:** 1 Department of Pathology, The Fourth Affiliated Hospital of Harbin Medical University, Harbin, China;; 2 Department of General Surgery, The Fourth Affiliated Hospital of Harbin Medical University, Harbin, China

**Keywords:** Breast cancer, lysosome, prognostic models, immune cell infiltration, EIF4EBP1, BRCA patients

## Abstract

**Background:**

Lysosomal dysfunction is significantly associated with tumor progression. This study aimed to identify and develop a new predictive panel for breast cancer (BRCA) and examine its relationship with the immune environment and therapeutical status.

**Methods:**

We developed a prognostic panel employing lysosomal genes from The Cancer Genome Atlas Program (TCGA) and then validated and assessed it externally in the Gene Expression Omnibus (GEO). Furthermore, the disparities were identified between high and low-risk subgroups by examining the infiltration of microenvironment cells, gene expression of immune checkpoints, and small molecular compounds. Ultimately, the cancerous function and potential pathway of core LRG were verified using a series of *in vitro* tests.

**Results and Discussion:**

First, the predictive panel of lysosome-related genes (LRGs) was generated *via* the least absolute shrinkage and selection operator. High-risk populations showed the shortest survival times. Meanwhile, the area under the curves (AUC) for predicting 1-, 3-, and 5-year survival rates indicated good predictive performance across all cohorts. Subsequent extensive investigations revealed a strong correlation between the risk score and the pathological stage, drug sensitivity, and tumor mutation burden (TMB). Then, we discovered that the levels of GPLD1, PLA2G5, and STX7 were reduced in BRCA tissues, whereas the expressions of PLA2G10, LAMP3, EIF4EBP1, and LPCAT1 were elevated in BRCA tissues compared to paracancerous tissues. Patients exhibiting high EIF4EBP1 expression experienced a more unfavorable outcome compared to those with low expression. EIF4EBP1 disruption dramatically impeded BRCA cell growth and invasive capacity, as demonstrated by CCK8, wound healing, and transwell assays. Moreover, EIF4EBP1 silencing in BRCA cells significantly restricted the TGF-β pathway.

**Conclusion:**

Our 9-LRG panel is a promising classifier for assessing the prognosis of BRCA. Notably, targeting EIF4EBP1 could potentially serve as a theoretical foundation for enhancing the prognosis of BRCA patients.

## 1 INTRODUCTION

With the greatest incidence rate and fifth-highest mortality rate in the world, breast cancer (BRCA) is one of the most frequent cancers in women [1-3]. Depending on the clinical tumor subtype, the primary treatment options for BRCA include chemotherapy, anti-HER2 targeting, and endocrine therapy [4, 5]. Although BRCA incidence has recently increased, the mortality rate has been greatly reduced due to more accurate early screening and more targeted treatment [6, 7]. However, patients with advanced BRCA have poor prognoses. A study revealed that just 29% of stage IV patients survived for five years [8]. Hence, it is imperative to discover the latent molecular pathways that contribute to the development of cancer and identify novel prognostic markers to enhance the clinical outcomes of individuals with BRCA.

Lysosomes are membrane-enclosed cytoplasmic acidic organelles that regulate cellular and organismal homeostasis. Lysosomes also degrade biomolecules through various lysosomal hydrolases [9-11]. Besides participating in autophagy and multiple cell death pathways, lysosomes regulate nutritional perception and immune response [12, 13]. In addition, emerging evidence has indicated that lysosomes can act as the signaling, metabolic, and quality control center for cells that modulate cell growth, division, and differentiation [14]. Multiple studies have demonstrated that alterations and impairment in lysosomes can impact the progression of various human ailments, such as malignancies, neurological disorders, and cardiovascular diseases [15]. Furthermore, lysosome dysfunction and structural abnormality can promote the progression of BRCA, and thus serve as therapeutic targets for suppressing tumors. Meanwhile, the downregulation of NAD-dependent deacetylase Sirtuin 1 can promote BRCA cell growth and invasion by disrupting lysosomal function and altering the secretome of BRCA cells [16]. LAMP3 upregulation can improve tamoxifen chemoresistance in BRCA cells by regulating autophagy [17]. Moreover, hexamethylene amiloride induces ROS and lysosome-mediated forms of programmed necrosis, thereby selectively targeting heterogeneous cancer cell populations in BRCA [18]. Besides, ursolic acid derivative UA232 induces lysosomal dysfunction, resulting in lysosome-dependent cell death in BRCA cells [19]. These studies illustrate that targeting lysosomes might be a useful BRCA treatment strategy.

Recent years have witnessed significant progress in the development of molecular prognostic panels for BRCA, leveraging multi-omics approaches to decode tumor heterogeneity. Several prognostic signatures are based on distinct biological processes—including cuproptosis, ferroptosis, and necroptosis [20-22]. Additionally, emerging methodologies, such as single-cell RNA sequencing and spatial transcriptomics, further refine prognostic accuracy by resolving intratumoral heterogeneity and identifying rare subpopulations associated with metastasis [23, 24]. Although a recent study employed machine learning to combine mitochondrial and lysosomal genes for the prognosis evaluation of BRCA patients [25]. However, the comprehensive integration of lysosomal genomic features for breast cancer prognosis prediction and the validation of the role of core genes in BRCA progression remain largely unexplored.

In this work, a panel containing nine lysosome-related genes (LRGs) was established for BRCA patients and discovered that LRG was an independent prognostic indicator. Meanwhile, multiple GEO datasets were used for verification. The functional characteristics of different risk subgroups were analyzed in multiple aspects based on LRGs. The hub LRG was characterized *via* immunohistochemistry using the HPA database. Ultimately, the oncogenic functions of EIF4EBP1 were ascertained by *in vitro* tests.

## 2 MATERIALS AND METHODS

Our study employs a systems biology framework to integrate multi-omics datasets, thereby elucidating the role of lysosome-associated genomic features in the prognostic evaluation and molecular underpinnings of BRCA. The methodologies central to our workflow include:

### 2.1 Multi-Omics Data Integration

We acquired transcriptome, mutation data, and clinical information from The Cancer Genome Atlas (TCGA), which consisted of 113 normal tissues and 1113 BRCA tissues. Three independent BRCA cohorts (GSE20711+ GSE96058+GSE42568, including 3273 samples) were collected from the Gene Expression Omnibus (GEO). Patients with a follow-up period of less than one month were excluded. The LRGs from the Gene Ontology Resource are shown in Table **S1**.

Ten pairs of BRCA and adjacent tissue specimens resected from the Department of Pathology of the Fourth Affiliated Hospital of Harbin Medical University between July 2018 and October 2021 were obtained. The hospital's ethics committee approved this study (No.202106). Informed consent was obtained from all patients before tissue samples were collected. Specimens were stored at -80 °C for until analysis.

### 2.2 Identification of Differential LRGs and Development of a Predictive Model

Differentially expressed genes (DEGs) in paracancerous and BRCA tissues were screened for using the “limma” package (FDR < 0.05 and │log2FC│≥ 1). The DEGs were then intersected with the LRGs to obtain differentially expressed LRGs. Significant prognostic value of LRGs was revealed by univariate Cox regression analysis.

A predictive panel related to LRGs was subsequently created using the least absolute shrinkage and selection operator (LASSO) algorithm, which was applied to reduce dimensionality and select the most predictive LRGs while avoiding overfitting. This method optimizes the trade-off between model complexity and predictive accuracy through penalized regression. Then, a scoring system was employed to calculate the risk scores for each patient with BRCA. The patients with BRCA were categorized into high-risk and low-risk subgroups according to the median score. Meanwhile, prognostic performance was evaluated using Kaplan-Meier (KM) and area under the curve (AUC) analysis.

### 2.3 Panel Robustness and External Validation

First, the risk scores for different clinical parameters, including age, stage, ER, PR, HER2, metastatic and recurrence, and chemoradiotherapy status, were investigated. The predictive performance of the LRGs-related prognostic panel was evaluated using a calibration curve and nomogram. Furthermore, independent prognostic indicators were identified based on the integrated clinicopathological features. Finally, the clinical characteristics of different risk patients were evaluated using a heatmap and KM curves.

Three independent GEO cohorts (nGSE20711=88, nGSE42568=104, and nGSE96058=3273) were used to externally validate panel stability. The chip data were standardized before the evaluation of prognostic performance. In addition, the protein expression patterns of the crucial LRGs were compared using the Human Protein Atlas (HPA).

### 2.4 Tumor Immune Microenvironment Evaluation

The Single-sample gene set enrichment analysis (ssGSEA) was implemented to calculate the ratios of immune cells in each patient. The relationship between immune cells and risk score was determined using seven algorithms (CIBERSORT, CIBERSORT-ABS, XCELL, EPIC, TIMER, MCPCOUNTER, and QUANTISEQ). These tools deconvolute bulk RNA-seq data into cell-type-specific signatures, enabling inference of tumor-immune interactions.

A mutation status analysis was conducted for the first 15 genes that were most commonly mutated between low- and high-risk patients using mutation data from TCGA to assess the clinical value of prognostic features of LRGs. The “survival” and “survminer” packages were utilized to evaluate the optimal threshold for tumor mutational burden (TMB) and conduct a Kaplan-Meier survival analysis in conjunction with the risk score.

Furthermore, the BRCA patients were classified according to various immune subtypes, as previously described [26]. Patients with immune checkpoint blockade have poor clinical outcomes. Therefore, ESTIMATE (A method for inferring the ratio of mesenchymal stromal cells to immune cells in a tumor sample using a gene expression signature) scores assessed stromal and immune contributions to tumor purity [27], while tumor immune dysfunction and exclusion (TIDE) algorithms were employed to predict immunotherapy response [28].

### 2.5 Prediction of Small Molecular Compounds

Various chemotherapeutic drugs, including Dihydrorotenone, Mitoxantrone, and Ribociclib, were predicted using the “pRRophetic” package. The Wilcoxon test was adopted to compare the inhibitory concentration (IC_50_) between different risk populations. Drug response predictions were generated using Genomics of Drug Sensitivity in Cancer (GDSC) data, linking risk subgroups to chemotherapeutic vulnerabilities.

### 2.6 Gene Set Variation Analysis

The co-associated genes of EIF4EBP1 were identified using the Pearson method to further reveal the biological function of EIF4EBP1. The “GSVA” package was utilized to investigate the overall manifestation pathways in various risk subgroups. Specifically, we first utilized the gene expression profiles of the TCGA-BRCA cohort, following the method outlined by Hänzelmann *et al*. [29]. We predefined the hallmark gene sets to evaluate the enrichment scores by setting the minimum gene set size to 5 and the maximum gene set size to 5000, and computed the pathway activity scores for gene sets to link LRGs to biological processes.

### 2.7 Cell Lines and Cell Culture

Cells, including MCF-10A, MCF-7, MDA-MB-468, BT-549, and MCF-231, were obtained from the Chinese Academy of Sciences' Cell Bank in Shanghai, China. The cells were cultured in DMEM (Gibco, USA) with 10% fetal bovine serum (FBS) (Biology Industries, Israel) at 37 °C and 5% CO2. These cell lines have been verified using STR profiling and reported negative for mycoplasma.

### 2.8 Plasmid Synthesis and Transfection

First, the siRNA sequence of human EIF4EBP1 was identified. DNA template oligonucleotides with three different siRNA sequences (siRNA-1 and siRNA-2) were then synthesized following the design guidelines of siRNA (Table **S2**). The siRNA and blank sequences were produced by Genechem Co., Ltd. The transfection procedure was carried out when the cultured BRCA cells reached a fusion degree of roughly 90%. Lipofectamine 3000 (Invitrogen) was selected for transfection, following the directions provided by the manufacturer, with NC, siRNA-1, and siRNA-2 plasmids.

### 2.9 Total RNA Extraction and Quantitative Real-time Polymerase Chain Reaction (qRT-PCR)

The TRIZOL reagent (Invitrogen) was implemented to extract the total RNA. cDNA was synthesized using the Toyobo (FSQ-101) kit. Adhering to the guidelines provided by the manufacturer. The qRT-PCR experiments were conducted using SYBR Green Master Mix (Vazyme) and GAPDH served as the internal reference. Table **S2** presents the sequences.

### 2.10 Cell Growth and Proliferation Assay

BRCA cells launched into 96-well plates, with each well reaching 30% confluence. A volume of 10 ul of CCK-8 reagent (CK04, Dojindo) was added to each well at one, two, and three days after culture, and incubated for 2 hours. The optical density was quantified at an intensity of 450 nm using an enzyme-labeled instrument (SynergyHTX, BioTek).

### 2.11 Wound-healing Assay

GC cells in the logarithmic growth phase were placed in a 6-well plate. Scratches were then created using a pipette tip perpendicular to the parallel lines. The floaters were sucked out after washing with PBS, while the remaining cells were cultured in 1% FBS medium for 48 hours. The samples were imaged (100 ×) using a microscope (Olympus X71, JPN) at specific intervals after cell separation.

### 2.12 Transwell Migration Assay

BRCA cells were reconstituted as single cells in a serum-free medium after 24 hours of transfection. A total of 2 x 10^5 cells (200 μL/well) were introduced into the upper chamber, while the lower chamber was filled with medium containing 20% FBS. The upper chambers were cleansed with PBS, followed by treatment with 4% paraformaldehyde for fixation. Afterward, cells were stained with a 0.1% aqueous solution of crystal violet after a 15 min period. The cells were removed from the surface of the polycarbonate membrane using a cotton swab. Finally, the cells penetrating the membrane were observed and counted using a microscope (Olympus X71, JPN).

### 2.13 Western Blot Analysis

First, RIPA Buffer (100 μL), phosphatase inhibitor A, and phosphatase inhibitor B (1:1 ratio, Beyotime Biotechnology) were added after collecting the cells. Protein concentration was quantified using the BCA method. The protein samples underwent electrophoresis using a 10% SDS-PAGE gel. Subsequently, they were transferred to Nitrocellulose membranes and sealed with milk for 2 hours. Following this, the membranes were incubated with the appropriate primary antibodies at a temperature of 4°C overnight. The nitrocellulose membrane was then incubated with appropriate HRP-conjugated secondary antibody at room temperature for two hours, followed by ChemiDoc XRS+ development (Bio-Rad Laboratories). Primary antibodies included antibodies against Smad2/3 (#AF6367; Affinity), Phospho-Smad2/3 (Thr8) (#AF3367; Affinity), TGF beta 1 (#a AF1027; Affinity), GAPDH (#AF7021; Affinity).

## 3 RESULTS

### 3.1 Identification of Differential Genes and Selection of Hub LRGs

**Fig. (1):**
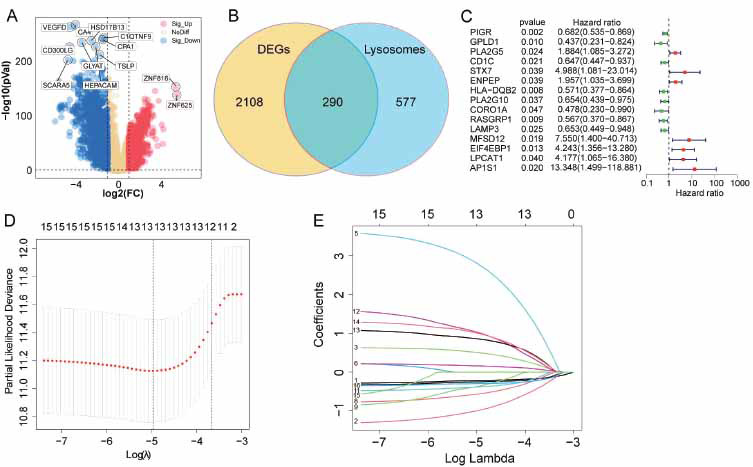
Differential expression analysis and prognostic hub LRG selection. (**A**) A volcano plot of differentially expressed genes between normal tissues and BRCA tissues. (**B**) Venn diagram showing differentially expressed LRGs. (**C**) Univariate Cox regression analysis was conducted to examine the differential LRGs associated with prognosis. *p*<0.05 represent significant differences. (**D**) Specific λ selection *via* 10-fold cross-validation. (**E**) The lambda parameter represents the coefficients of the selected features.

A total of 2398 genes exhibiting differential expression were identified, with 896 genes up-regulated and 1502 genes down-regulated, when comparing BRCA tissues to adjacent tissues (Fig. **1A** and Table **S3**). Furthermore, a total of 290 differentially expressed LRGs were found (Fig. **1B**). Prognostic significance of 15 LRGs was examined using univariate Cox regression (Fig. **1C**), where eight were protective factors (PIGR, GPLD1, CD1C, HLA-DQB2, PLA2G10, CORO1A, RASGRP1, and LAMP3), while the remaining seven were risk factors (PLA2G5, STX7, ENPEP, MFSD12, EIF4EBP1, LPCAT1, AP1S1). Hub LRGs were selected using the LASSO algorithm. The ultimate prognostic model for BRCA patients was established by choosing the optimal combination (Fig. **1D** and **E**).

### 3.2 Establishment of Risk Scores in BRCA Patients using the TCGA Set

**Fig. (2):**
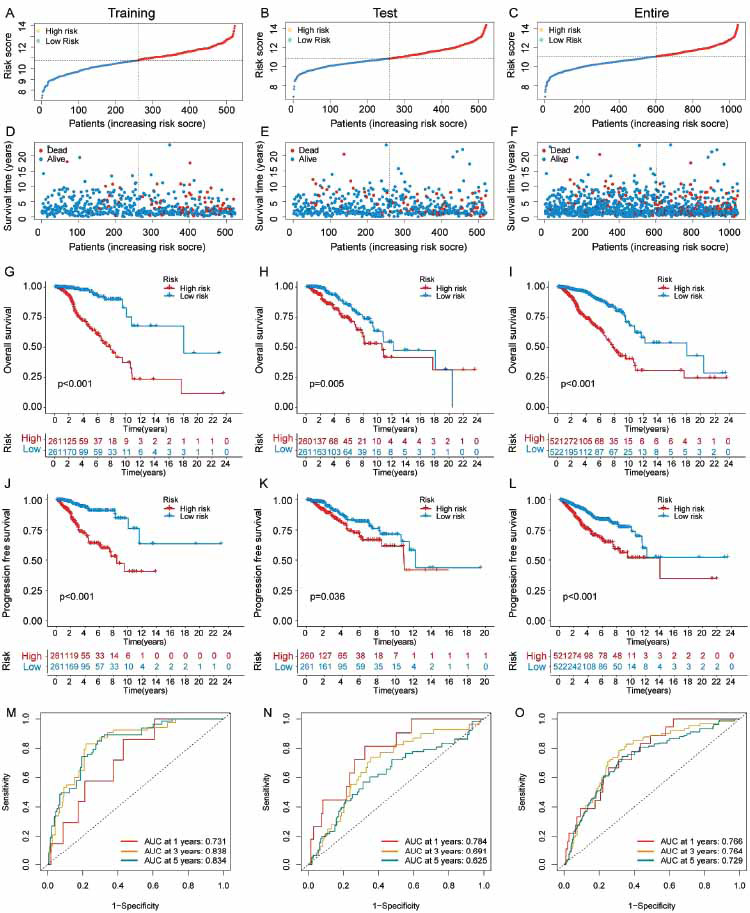
Developing a predictive panel based on LRGs. (**A-C**) Distribution of high and low-risk scores and survival status in training, test, and entire cohorts. (**D-F**) A scatter plot displaying the distribution of the survival time corresponding to risk scores of different samples. The Kaplan-Meier curves depict the survival probabilities of two subgroups in the training, test, and entire cohorts, respectively. (**G-I**) OS. (**J-L**) DFS. ROC curves for predicting 1-, 3-, and 5-year OS in training (**M**), test (**N**), and entire cohort (**O**).

The expression values of each LRG and risk coefficients were integrated to construct the model. Patients with BRCA were also divided into two groups in the TCGA cohort: a training group of 522 and a test group of 521. Fig. (**2A-F**) depicts the relationship between survival status and time across all datasets. Patients classified in low-risk groups showed a longer overall survival rate in the KM analysis (Fig. **2G-I**). Furthermore, high-risk populations had the worst progression-free survival (Fig. **2J-L**). Importantly, AUCs for the 1, 3, and 5 years were 0.731, 0.838, and 0.834 for the training cohorts, respectively (Fig. **2M**), 0.784, 0.691, and 0.625 for the test cohort, respectively (Fig. **2N**) and 0.766, 0.764, and 0.729 for the entire cohort, respectively (Fig. **2O**).

### 3.3 Verification of Prognostic Panel Using Independent GEO Cohort

**Fig. (3):**
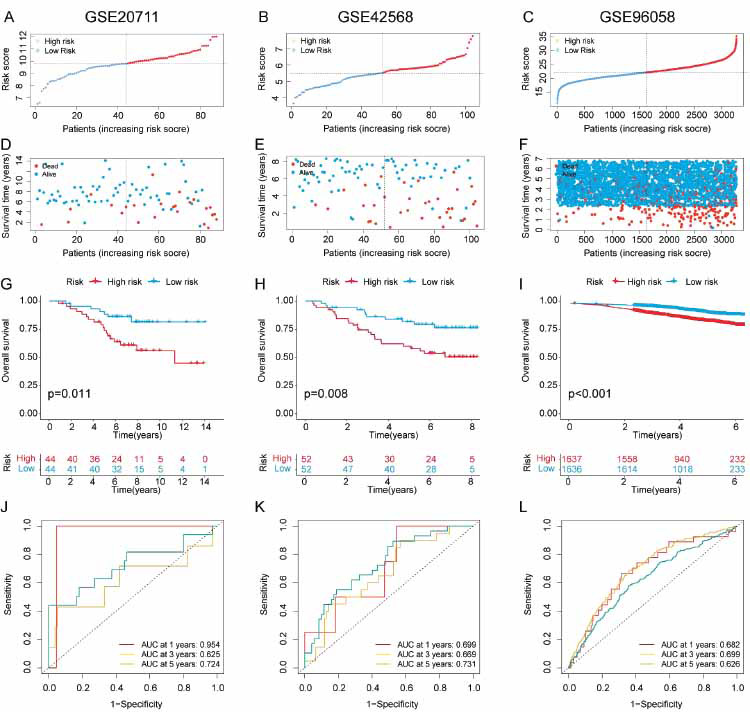
Verification of LRG-based panel in the GEO. A scatter plot representing the distribution of risk scores and survival status in the GSE20711 (**A**), GSE42568 (**B**), and GSE96058 (**C**). The survival time and distribution of risk scores in the GSE20711 (**D**), GSE42568 (**E**), and GSE96058 (**F**). KM analysis of the OS of two subgroups in GSE20711 (**G**), GSE42568 (**H**), and GSE96058 (**I**), respectively. ROC curves for predicting 1-, 3-, and 5-year OS in GSE20711 (**J**), GSE42568 (**K**), and GSE96058 (**L**).

The prognostic performance of the 9-LRG panel was assessed using three independent GEO cohorts (3465 BRCA patients). Mortality and risk scores were positively correlated (Fig. **3A-F**), implying that high-risk groups had shorter survival durations (Fig. **3G-I**). The AUCs at 1-, 3-, and 5 years were 0.954,0.625, and 0.724 for the GSE20711 cohort, respectively, 0.699,0.669, and 0.731 for the GSE42568 cohort, respectively, and 0.682, 0.699, and 0.66 for the GSE96058 cohort, respectively (Fig. **3J-L**). Our findings indicate that the LRG panel exhibited good stability and accuracy in the prognostic prediction of BRCA patients.

### 3.4 Clinicopathological Characteristics and Risk Score

**Fig. (4):**
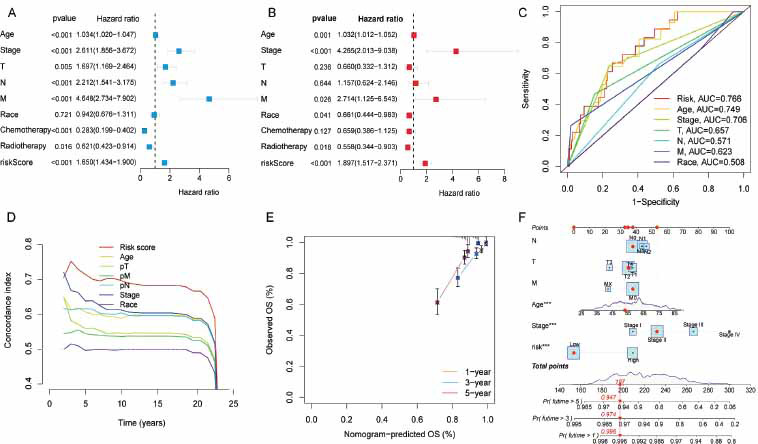
Evaluation of the prognostic performance of the 9-LRG panel. The forest map represents the correlation between the risk score and clinical parameters. (**A**) Univariate Cox analysis. (**B**) Multivariate Cox analysis. (**C**) Comparison of AUC between risk score and other clinicopathological parameters. (**D**) Comparison of concordance index (C-index) between risk score and other clinicopathological parameters. (**E**) Calibration curves of nomograms for predicting OS. (**F**) Nomogram integrating clinicopathological features and risk scores to forecast the possibility of survival time for BRCA.

The clinical values of a risk score for BRCA patients were also evaluated. Univariate Cox regression indicated that risk scores were related to survival (*p* < 0.001; Fig. **4A**). The information regarding the clinicopathological grouping was presented in Table **S4**. Furthermore, the risk score was identified as an independent risk prognostic indicator after adjusting for other confounders (Fig. **4B**). Moreover, the panel performed better at predicting BRCA prognosis than other clinical parameters based on AUC analysis (Fig. **4C**). The concordance index (C-index) demonstrated that the prognostic panel exhibited superior predictive accuracy compared to other clinical variables (Fig. **4D**). Meanwhile, the multivariable clinical parameters were used to construct nomograms for further prognosis prediction. The corresponding score for each variable was calculated separately, and the total score was used as a prognostic predictor for BRCA patients. Calibration curves confirm the accuracy of the predictions (Fig. **4E** and **F**).

Additionally, a heatmap of risk scores and clinical variables demonstrated that there were significant differences between the various risk groups in terms of T, M, and Estrogen receptor (ER), chemotherapy, as well as relapse and metastasis (Fig. **S1A**). KM results also indicated that the high-risk population for all variables had a lower survival rate based on each clinical feature. Previous analyses showed similar results (Fig. **S1B-I**).

### 3.5 Immune Cell Infiltration is Associated with the Risk Panel

**Fig. (5):**
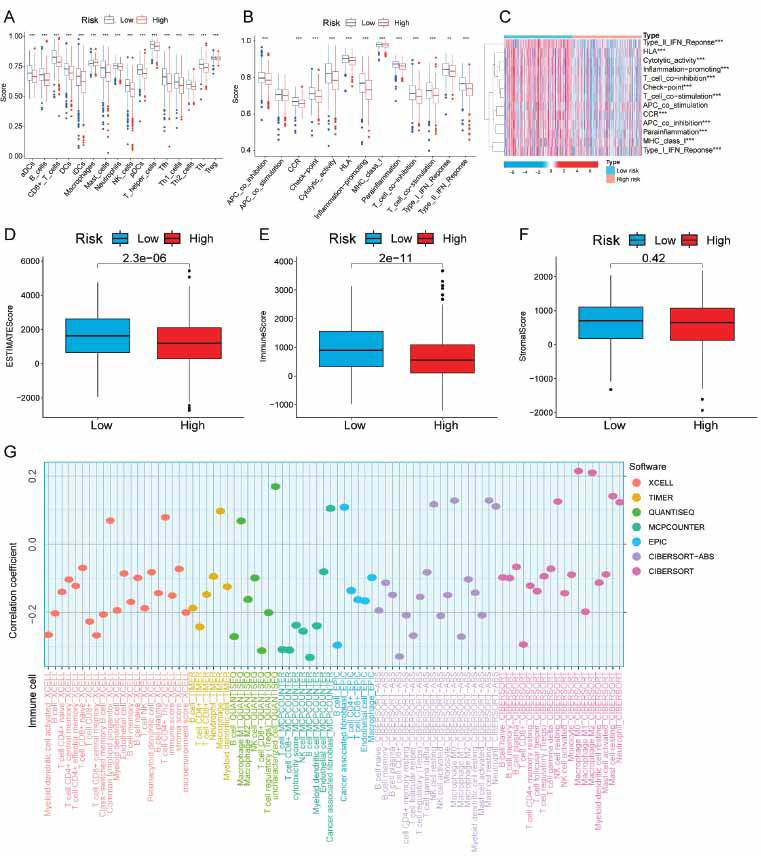
Risk panel and immune cell infiltration analysis. (**A**) A box plot illustrating the risk scores of 16 immune cells in patients. (**B** and **C**) A box plot and heatmap are employed to visualize the risk scores and enrichment degree of 13 immune-related functions in patients with varying risk scores. Asterisks (*) indicate statistically significant differences, with * representing *p* < 0.05, ** representing *p* < 0.01, and *** representing *p* < 0.001. (**D-F**) The box plot represents the immune scores, stromal scores, and estimate scores for both the high-risk and low-risk populations. (**G**) The association between risk score and infiltrating immune cells was assessed with 7 different methods.

SsGSEA analysis illustrated a greater presence of low-risk immune cells compared to high-risk immune cells in terms of infiltration (Fig. **5A**). Further, 12 distinct immune-related signaling pathways exhibited differences between the two subgroups (Fig. **5B** and **C**). To predict non-tumor cell infiltration, we employed the ESTIMATE to analyze immune and stromal cell gene expression. Participants classified as low-risk reported higher ESTIMATE scores and immune scores compared to participants classified as high-risk (Fig. **5D** and **E**). Nevertheless, there was no significant disparity in stromal scores between the two subgroups (Fig. **5F**). The immune infiltration ratios of 22 cells were then assessed using seven common algorithms. Two algorithms (MCP-counter and xCell) were based on the expression of 9 LRGs, while the remaining five were based on the deconvolution algorithm. Our findings demonstrated a link between immune cells and risk scores (Fig. **5G**).

### 3.6 Correlation between Risk Scores and Immunotherapy

**Fig. (6):**
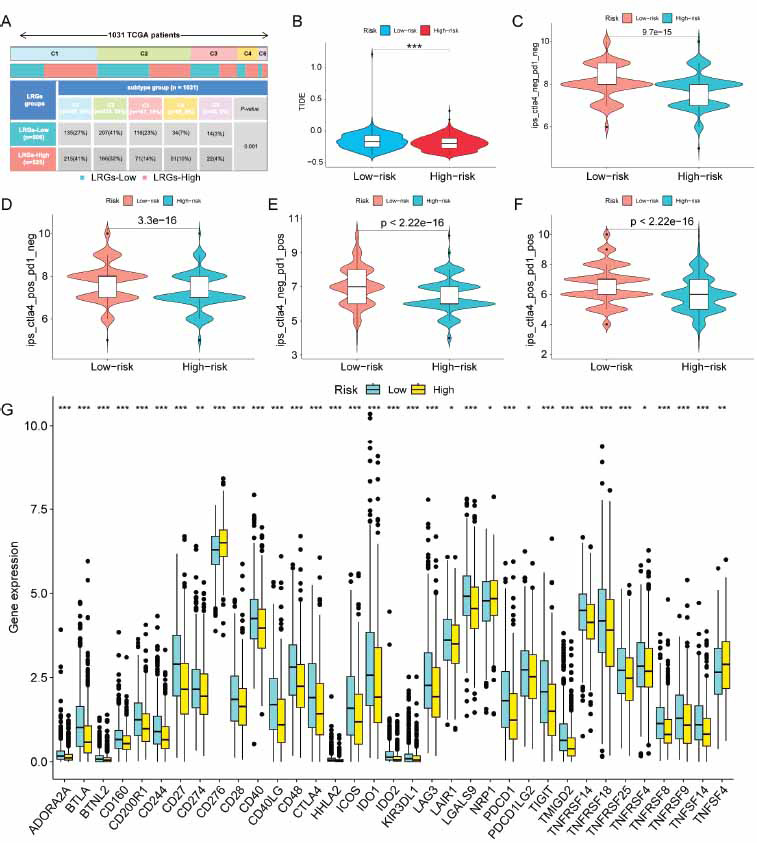
The risk score and immune response prediction. (**A**) Bar chart showing the proportion of immunologic subtypes in the different risk groups. (**B**) Violin plots depicting the disparities in TIDE scores between two populations at different risk levels. Immunophenotypic (IPS) differences in ICB treatment between patients with various risk scores; (**C**) ips_ctla4_neg_pd1_pos, (**D**) ips_ctla4_pos_pd1_neg, (**E**) ips_ctla4_neg_pd1_pos, (**F**) ips_ctla4_pos_pd1_pos. (**G**) Analysis of the expression levels of 35 immune checkpoint genes in populations of two subgroups. Asterisks (*) indicate statistically significant differences, with * representing *p* < 0.05, ** representing *p* < 0.01, and *** representing *p* < 0.001.

Tumor heterogeneity is a key factor contributing to unfavorable prognosis and the recurrence of BRCA [30]. Patients in the low-risk group in this study had C3 immune subtypes, while patients with C1, C4, and C6 immune subtypes made up the majority of the high-risk group (Fig. **6A**). A further point to consider is that the population at high risk had a lower TIDE score than the group at low risk (Fig. **6B-F**), indicating that CTLA4 and PD1 immunotherapy may significantly benefit the low-risk patients. Further comparison analysis showed that most common immune checkpoint genes were downregulated in high-risk patients, with only CD276 and TNFSF4 being highly expressed (Fig. **6G**). Therefore, targeting these two immune checkpoint genes may improve the immunotherapy efficacy of high-risk groups.

### 3.7 Identification of Latent Drugs for BRCA Patients

**Fig. (7):**
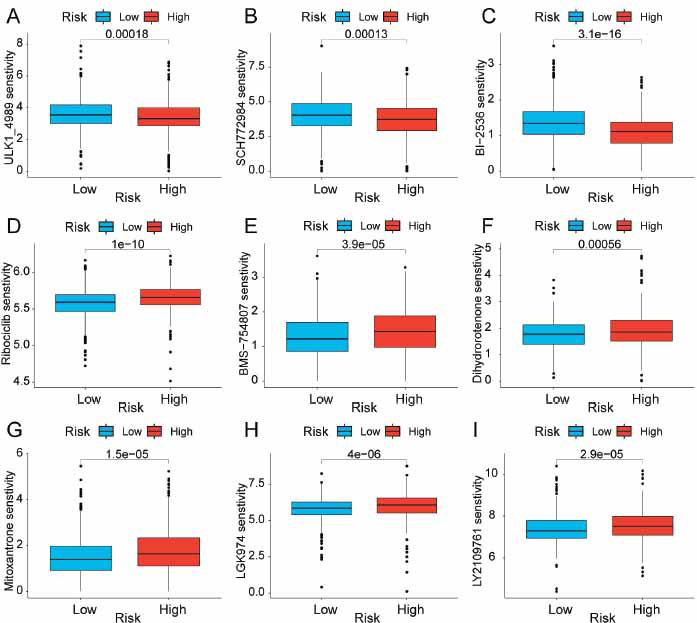
Association of 9-LRG prognostic signature with drug sensitivity. Box plots presenting the IC_50_ values of potential compounds in two subgroups of BRCA patients. (**A**) ULK1_4989, (**B**) SCH772984, (**C**) B1-2536, (**D**) Ribociclib, (**E**) BMS-754807, (**F**) Dihydrorotenone, (**G**) Mitoxantrone, (**H**) LGK974, (**I**) LY2109761.

Further analysis evaluated whether the 9-LRG panel can predict the sensitivity of a group of BRCA patients to several different agents to further enhance the therapeutic effect of neoadjuvant chemotherapy. The IC_50_ value of each small molecule was determined based on the GDSC database. Various potential compounds related to BRCA treatment were screened. Results found that high-risk populations had lower IC_50_ values of ULK1_4989, SCH772984, and B1-2536 (Fig. **7A-C**), while the low-risk population exhibited lower IC_50_ values of Ribociclib, BMS-754807, Dihydrorotenone, Mitoxantrone, LGK974, and LY2109761 (Fig. **7D-I**). These findings indicate that the developed panel has potential predictive value for individualized chemotherapy in different populations.

### 3.8 Hub LRGs Expressed in Normal and BRCA Tissues

**Fig. (8):**
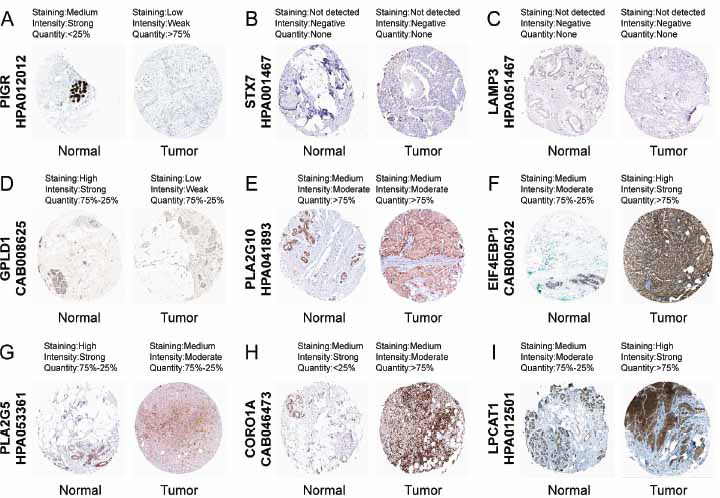
Protein expression levels of nine hub LRGs in normal and BRCA tissues were analyzed employing the HPA. (**A**) PIGR, (**B**) STX7, (**C**) LAMP3, (**D**) GPLD1, (**E**) PLA2G10, (**F**) EIF4EBP1, (**G**) PLA2G5, (**H**) CORO1A, (**I**) LPCAT1.

The HPA database was utilized to detect the protein levels of nine LRGs in both cancerous and normal tissues. Compared with normal breast tissues, PIGR, GPLD1, PLA2G5 and CORO1A were down-regulated in cancer tissues, while EIF4EBP1 and LPCAT1 were significantly up-regulated. However, STX7, PLA2G10, and LAMP3 expressions were not discernible differences between subgroups (Fig. **8**). The mRNA levels of these hub LRGs mRNA levels were detected *via* qRT-PCR. BRCA tissues had lower levels of PLA2G5, STX7, GPLD1, and PIGR mRNA than normal tissues (Fig. **S3A-C**), while mRNA expressions of LPCAT1, EIF4EBP1, PLA2G10, and LAMP3 increased (Fig. **S3D-G**). Nevertheless, there was no substantial disparity in the mRNA expression of CORO1A in subgroups, as depicted in Fig. (**S3H**).

### 3.9 Suppression of EIF4EBP1 Hampers the Growth, Migration, and Invasion of BRCA Cells

**Fig. (9):**
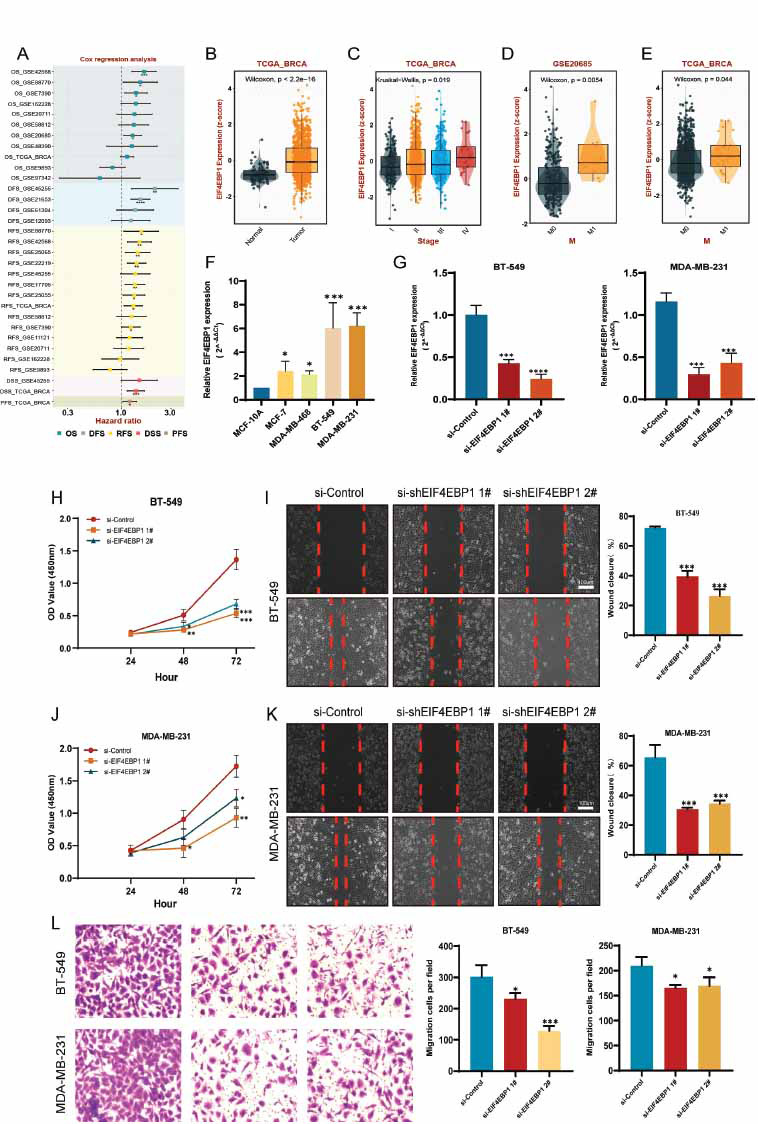
Disruption of EIF4EBP1 hindered the growth, migration, and invasion of BRCA cells. (**A**) COX regression analysis of EIF4EBP1 for GEO and TCGA multicohorts. (**B**) Comparison of EIF4EBP1 expression between normal and BRAC tissues by the Wilcoxon test. (**C**) Comparison of EIF4EBP1 expression among stages by the Kruskal-Wallis. (**D**) and (**E**) Comparison of EIF4EBP1 expression between M0 and M1 using the Wilcoxon test. (**F**) EIF4EBP1 expression was examined in both normal breast epithelial cells and BRCA cells. (**G**) The knockdown efficiency of BT-549 cells (the left bar chart) and MDA-MB-231 cells (the right bar chart). (**H**) CCK8 assay exhibits the cell growth ability of BT-549 cells. (**I**) The wound scratch assay reflects the BT-549 cell's migration capacity. (**J**) CCK8 assay exhibits the cell growth ability of MDA-MB-231 cells. (**K**) The wound scratch assay reflects the MDA-MB-231 cell's migration capacity. (**L**) Transwell assay indicating the invasive capacity of BT-549 cells (top half) and MDA-MB-231 cells (bottom half), respectively. Significant differences are represented by asterisks (*): * *p* < 0.05, ** *p* < 0.01, and *** *p* < 0.001.

Cox regression analysis suggested that EIF4EBP1 is a prognostic risk factor for BRCA patients (Fig. **9A**). EIF4EBP1 was substantially elevated in BRCA tissues and exhibited an association with stage and distant metastasis (Fig. **9B-E**). Further, EIF4EBP1 expression in normal breast epithelial cells and four cancer cells was detected using qRT-PCR. EIF4EBP1 had higher expression in BRCA cells than in normal epithelial cells. BT-549 and MDA-MB-231 cells were employed in the phenotyping studies that followed (Fig. **9F**). The knockdown efficiency after transfection of si-EIF4EBP1 is shown in Fig. (**9G**). CCK8 assay proved that transfection with si-EIF4EBP1 impaired the growth of BRCA cells (Fig. **9H** and **J**). EIF4EBP1 deletion restricted BRCA cell migration during wound healing, compared to the control group (Fig. **9I** and **K**). The Transwell experiment showed similar phenotypes. Notably, the invasion ability of both two cancer cells decreased significantly after infection with si-EIF4EBP1 (Fig. **9L**).

### 3.10 EIF4EBP1 Positively Regulates the TGF-β Signaling Pathway

**Fig. (10):**
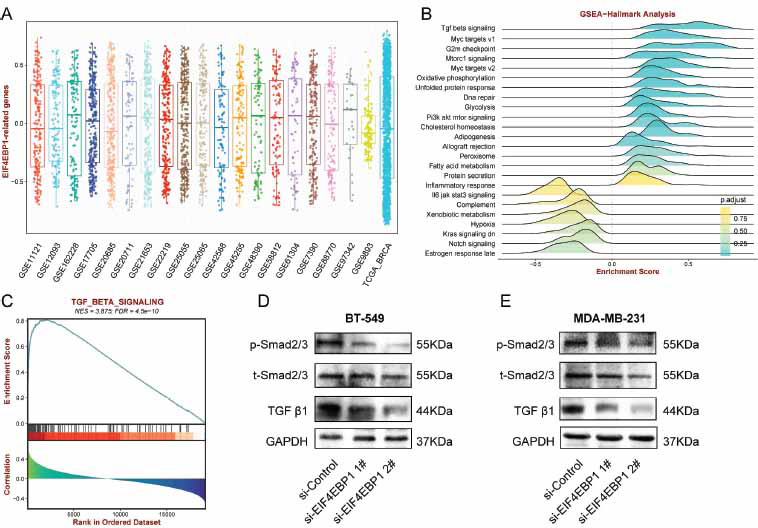
EIF4EBP1 positively mediates the TGF-beta signaling pathway. (**A**) The bar graph illustrating EIF4EBP1 normalization in multiple. (**B**) The ridge plot represents the GSVA analysis of the EIF4EBP1 co-associated genes. (**C**) A positive association was found between EIF4EBP1 and the TGF-β pathway. (**D**) Western blot detection of TGF-β and p-Smad2/3 expression in BT-549 cells. (**E**) Western blot detection of TGF-β and p-Smad2/3 expression in MDA-MB-231cells.

The co-associated genes with EIF4EBP1 were identified *via* Pearson correlation to further determine the latent downstream pathways of EIF4EBP1 (Table **S5**). GSVA analysis was conducted following the normalization of the GEO and TCGA (Fig. **10A**). The results confirmed a significant enrichment of these genes in the TGF-beta signaling pathway (Fig. **10B** and **C**). Following this, Following, the western blot results proved hampering EIF4EBP1 had a substantial inhibitory effect on the levels of TGF-β and p-smad2/3 (Fig. **10D** and **E**).

## 4 DISCUSSION

Despite significant advancements in surgical approaches, the overall clinical outcomes for patients with BRCA remain unsatisfactory. Genomic landscapes of various cancers have been elucidated using next-generation sequencing technologies [31, 32]. Nonetheless, BRCA patients lack effective prognostic biomarkers. Research discovered that the malfunctioning of lysosomes is linked to various human diseases, such as cancer [15, 33]. This study makes several significant contributions: First, the robustness of our constructed 9-LRG panel was confirmed through both an internal cohort and a large external cohort (n=3465). Second, multidimensional analysis showed that 9-LRG scores were associated with tumor-immune interactions and treatment resistance. Importantly, we emphasized the translational implications of targeting EIF4EBP1 and its downstream TGF-β pathway in BRCA management.

Patients with high- and low-risk BRCA could be distinguished by their risk score. The risk score was determined to be an independent prognostic indicator for BRCA. In addition, the nomogram and calibration curve demonstrated that the 9-LRG panel exhibited promising predictive capabilities. qRT-PCR analysis confirmed a significant increase in EIF4EBP1 expression in BRCA tissues as compared to normal tissues. Moreover, the elevated expression of EIF4EBP1 was linked to unfavorable outcomes in the KM analysis. Experimental analysis conducted in a laboratory setting showed that the suppression of EIF4EBP1 had a significant inhibitory effect on the growth and invasion of BRCA cells. Additionally, it also hindered the activity of the TGF-β pathway, thereby identifying potential targets for therapeutic intervention in BRCA.

Emerging evidence increasingly underscores the pivotal role of lysosomes in the progression of BRCA, highlighting their significance in tumor biology and therapeutic potential. The integrity of mTOR signaling, regulated by lysosomal TMEM9-LAMTOR4, has been identified as a critical determinant for breast tumorigenesis [34]. G3BP1 and G3BP2, key components of stress granules (SGs), are predominantly localized on lysosomal surfaces. In tumor contexts, reduced G3BP1 expression promotes mTORC1-mediated breast cancer cell motility and correlates with unfavorable clinical prognosis [35]. Furthermore, the lysosomal gene ATP6AP1 has been demonstrated to confer doxorubicin resistance in breast cancer through the upregulation of autophagic flux [36]. He *et al.* [37] revealed that mTORC1 suppression increases lysosomal acidification, facilitating lysosomal cyst(e)ine-mediated adaptation to oxidative stress in breast cancer cells. Encouragingly, the lysosome-targeted magnetic nanotorquer T7-MNT dynamically activates endogenous Fe2+ reservoirs, presenting a novel approach for individualized breast cancer therapy [38]. In this study, we identified nine core genes associated with lysosomal function. PIGR dysfunctions are associated with various precancerous lesions and tumors [39].

In this study, the expression of PIGR decreased substantially in BRCA tissues compared to normal tissues, both at the protein and mRNA levels. This decrease in expression may be attributed to the malignant progression of BRCA. However, elevated PIGR expression has been reported linked to unfavorable prognoses in several cancers [40, 41]. Research has clarified that M1 macrophages raised PIGR levels in BRCA cells employing IL-1β [42], suggesting that targeting M1 macrophages to increase PIGR may be a potential anti-tumor strategy. Furthermore, STX7 can mediate MDA-MB-231 cell invasion by interacting with STX4, thus forming multiple SNARE complexes [43]. A study showed that LAMP3 is induced by the PERK/ATF4 arm and is variably expressed in BRCA cell lines [44]. The PERK/ATF4/LAMP3-arm mediates hypoxia-induced cancer cell migration, indicating that inhibiting this axis can result in the radiosensitization of BRCA cells. Therefore, the PERK/ATF4/LAMP3 arm is a promising therapeutic target [45, 46]. LPCAT1 exhibits significant elevations in various types of cancer, including hepatocellular carcinoma [47], esophageal squamous cell carcinoma [48], and lung adenocarcinoma [49]. Notably, LPCAT1 is considered an oncogene. LPCAT1 knockdown can significantly inhibit cell growth and invasion. Voeltzke K *et al.* [50] discovered that MYCN transcription can up-regulate EIF4EBP1, thus promoting the poor prognosis of neuroblastoma. Besides, EIF4EBP1, as a carcinogenic regulator, can affect BRCA cell proliferation by mechanisms distinct from its regulation of cap-dependent translation [51]. In BRCA tissues and cell lines, EIF4EBP1 was highly expressed. Furthermore, EIF4EBP1 silencing significantly inhibited the malignancy progression of BRCA cells *in vitro*. Aberrant activation of the TGF-β signaling pathway significantly causes epithelial-mesenchymal transition [52]. Herein, GSVA analysis showed that EIF4EBP1 was strongly positively correlated with the TGF-β signaling pathway. Furthermore, EIF4EBP1 silencing significantly inhibited TGF-β pathways, suggesting that targeting EIF4EBP1 and TGF may offer therapeutic prospects for BRCA.

The molecular features of BRCA based on lysosomes are unknown. Therefore, complementary studies targeting lysosomes and regulating the components of immunosuppressive TME are crucial in the development of multiple solid tumor therapies. Dendritic cells and CD8+ T cells were infiltrated in low-risk groups in this work, whereas high-risk groups presented immunosuppressive microenvironment landscapes, explaining the treatment resistance in the patients. CTLA-4 and PD-1/PD-L1 are important clinical factors when targeting immune checkpoints (ICB) [53]. Patients at low risk benefit more from single or combined CTLA-4/PD-1 targeting than patients at high risk. Although patients who receive ICB therapy can benefit from the therapy for a long time, only a small percentage respond to the treatment over time [28, 54]. Herein, TIDE discovered that low-risk populations exhibited higher TIDE scores than high-risk populations. Our findings may improve personalized and targeted therapy strategies for different risk panels.

Furthermore, diverse chemotherapy drugs showed significantly different sensitivity in the two groups. Evidence has shown that the progression of BRCA is affected by Unc-51-like kinase 1 (ULK1) [55, 56]. Further investigation has also revealed that molecular agonists targeting ULK1 may become future treatments for BRCA [57, 58]. We observed that ULK1_4989 had a lower IC_50_ value in the high-risk populations, suggesting that it is suitable for BRCA treatment. Ribociclib, a CDK4/6 inhibitor, can treat BRCA patients by prolonging the median survival of metastatic patients with HR^+^/HER2^-^ [59]. Additionally, Mitoxantrone, as a topoisomerase II inhibitor, has a broad-spectrum anti-tumor effect and often works synergistically with other anti-tumor drugs [60, 61]. The IC_50_ of the above drugs was markedly lower in low-risk patients compared to high-risk individuals, indicating that low-risk individuals may exhibit greater sensitivity to these drugs.

## STUDY LIMITATIONS

Nevertheless, this study has some limitations. First, future studies incorporating multi-center, real-world clinical data are warranted to further validate the robustness and generalizability of our findings. Second, the predictive performance of the panel in the cohort was not assessed due to the lack of sufficient follow-up information. Third, the role of EIF4EBP1 in BRCA progression was preliminarily validated using *in vitro* experiments. Therefore, further *in vitro* and *in vivo* studies should evaluate the role of EIF4EBP1 and the remaining LRG in BRCA progression.

## CONCLUSION

A novel panel of lysosomal-derived prognostic signatures was developed to predict outcomes in BRCA patients. The model can assess the microenvironmental landscape and immunotherapy status. This study provides methods for optimizing chemotherapy regimens in BRCA patients. Importantly, the carcinogenic role of EIF4EBP1 in BRCA has been preliminarily investigated, providing a new rationale for targeted intervention strategies.

## Data Availability

All data are supporting the conclusions of this work included in the article and its supplementary files. Public data are from TCGA (https://portal.gdc.cancer.gov/), GEO (https://www.ncbi.nlm.nih.gov/geo/), Gene Ontology Resource (http://geneontology.org/), HPA (https://www.proteinatlas.org/), GDSC (https://www.cancerrxgene.org/).

## LIST OF ABBREVIATIONS

AUC: Area Under the Curves
BRCA: Breast Cancer
C-index: Concordance Index
DEGs: Differentially Expressed Genes
ER: Estrogen Receptor
FBS: Fetal Bovine Serum
GDSC: Genomics of Drug Sensitivity in Cancer
GEO: Gene Expression Omnibus
HPA: Human Protein Atlas
KM: Kaplan-Meier
LASSO: Least Absolute Shrinkage and Selection Operator
LRGs: Lysosome-related Genes
SGs: Stress Granules
ssGSEA: The Single-sample Gene Set Enrichment Analysis
TCGA: The Cancer Genome Atlas Program
TIDE: Tumor Immune Dysfunction and Exclusion
TMB: Tumor Mutation Burden
ULK1: Unc-51-like kinase 1
