# Supplementary material for: Development of a Novel Lysosomal Gene-based Prognostic Panel and Uncovering EIF4EBP1 as a Biomarker for Breast Cancer
Source: Curr Genomics. 2025 Jul 3;26(5):368–88. doi: 10.2174/0113892029357021250626210819 (PMC12728594; doi:10.2174/0113892029357021250626210819)
Supplement: Supplementary file 1 [file CG-26-5-368_SD1.zip › CG-26-5-368_SD1/CG-26-5-368_SD1.pdf]

Supplementary Material

Development of a Novel Lysosomal Gene-based Prognostic Panel and Uncovering EIF4EBP1 as a Biomarker for Breast Cancer

Bingkun Wang[1,#], Nianjin Wei[2,#], Meiyu He[1], Guocai Zhong[1] and Shujun Zhang[1,*]

[1]Department of Pathology, The Fourth Affiliated Hospital of Harbin Medical University, Harbin, China; [2]Department of General Surgery, The Fourth Affiliated Hospital of Harbin Medical University, Harbin, China

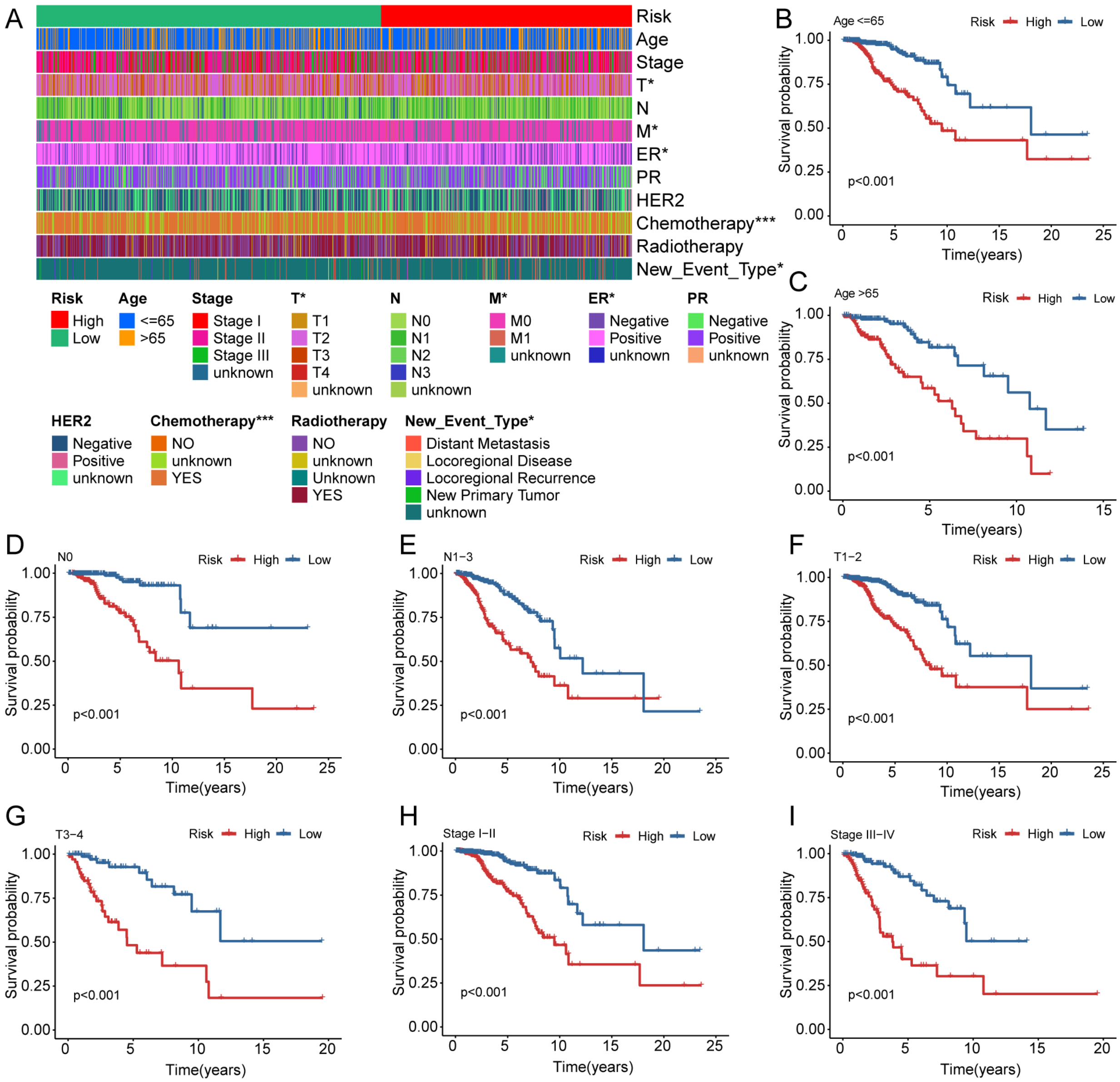

Supplementary Fig. (1). Clinical correlation analysis of LRG risk score panel. (**A**) The heatmap exhibits the connections between the risk score and other clinical parameters. Asterisks (*) denote statistically significant differences, with * indicating $p<0.05$, ** indicating $p<0.01$, and *** indicating $P < 0.001$. The KM curve illustrates differences between distinct ages (**B** and **C**), N states (**D** and **E**), T states (**F** and **G**), and stages (**H** and **I**) between high- and low-risk patients.

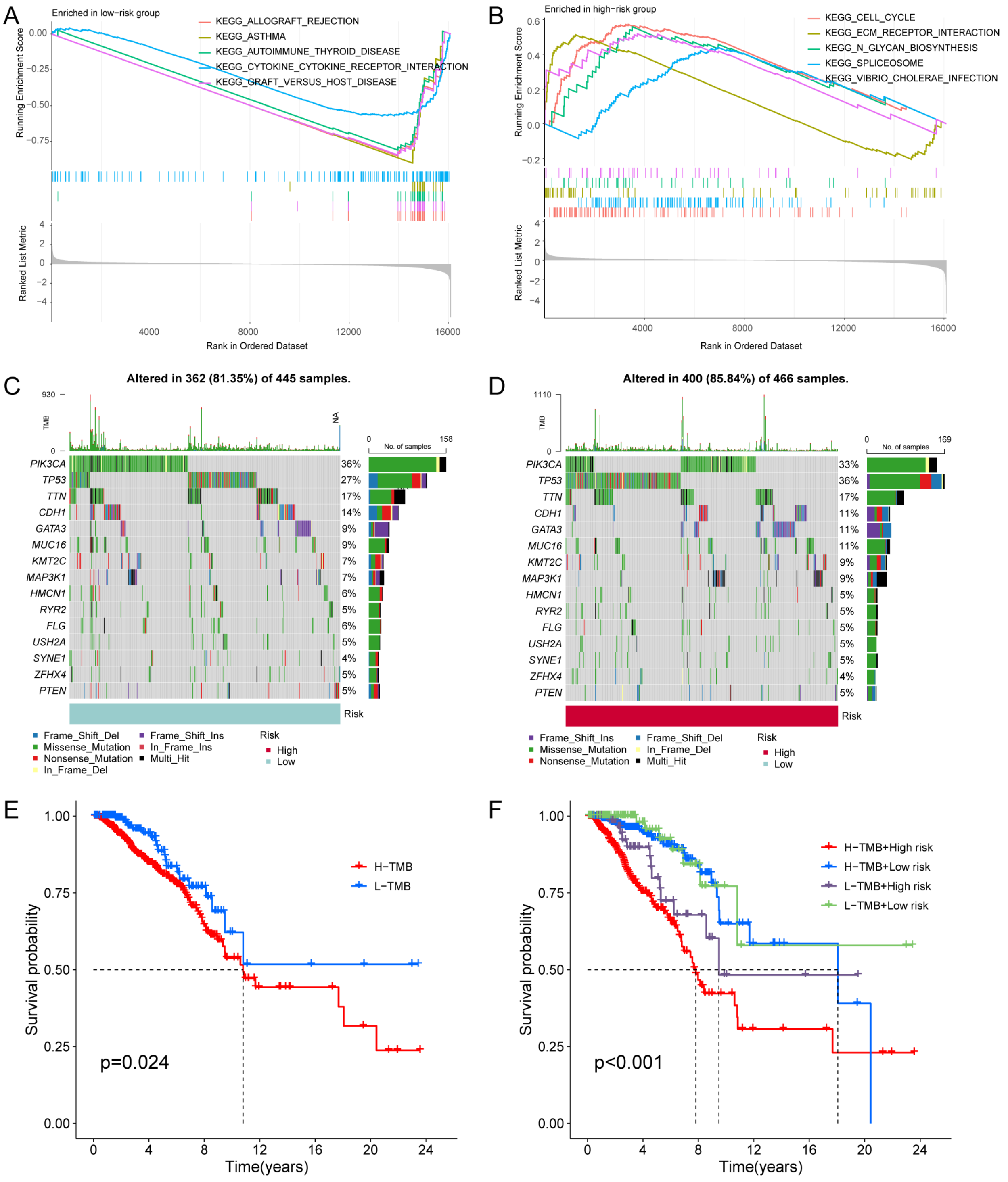

Supplementary Fig. (2). Analysis of functional characteristics between different risk groups. GSVA exhibited significant enrichment in the top five signaling pathways. (**A**) Low-risk group. (**B**) High-risk group. (**C**) Mutations in low-risk patients. (**D**) Mutations in high-risk patients. (**E**) KM curve presents the survival outcomes of populations with high TMB and low TMB. (**F**) KM curves indicate the survival of patients with TMB combined with a risk score.

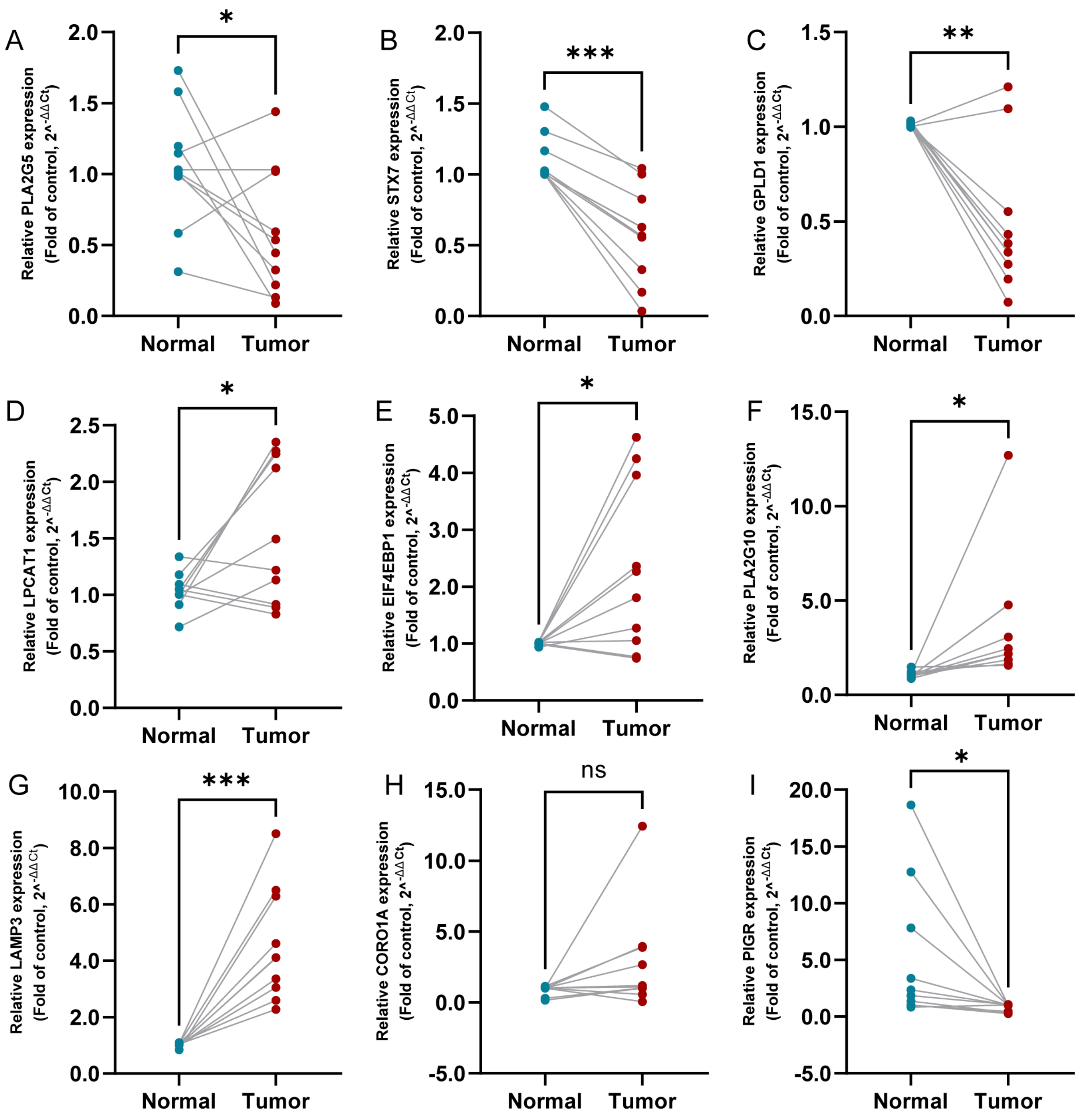

Supplementary Fig. (3). LRGs expression in-house cohort. The results of qRT-PCR detection of the expression of 9 hub LRGs in 10 pairs of normal tissues and BRCA tissues. (**A**) PLA2G5, (**B**) STX7, (**C**) GPLD1, (**D**) LPCAT1, (**E**) EIF4EBP1, (**F**) PLA2G10, (**G**) LAMP3, (**H**) CORO1A, (**I**) PIGR.
